# Rapamycin for the aging skin

**DOI:** 10.18632/aging.102664

**Published:** 2019-12-27

**Authors:** Mikhail V. Blagosklonny

**Affiliations:** 1 Cell Stress Biology, Roswell Park Cancer Institute, Buffalo, NY 14263, USA

**Keywords:** rapamycin, mTOR, cream, topical, anti-aging, cosmetics, patents

In 2007, I filed a patent application claiming that topical rapamycin (e.g., in the form of a cream or ointment) https://patents.google.com/patent/WO2008022256A2/en could be used to prevent and treat skin aging. Potential indications include various types of age-related spots, wrinkles, photo-aged skin, and other age-related skin conditions. The patent was not granted, nor were cosmetic companies interested in pursuing this avenue of product development. Cell senescence has traditionally been seen as growth arrest. It seemed weird that rapamycin, a drug that inhibits growth, could inhibit cellular senescence. Nonetheless, it works because, actually, senescence is a continuation of growth when true growth is impossible [1]; in other words, senescence is “twisted” growth [2]. In an exciting ‘twist’, these claims were recently confirmed in a clinical trial by Chung et al. [3], which I will discuss later.

Even in 2007, the idea of using rapamycin topically was not novel [4,5]. (What was novel in my application was the idea of using topical rapamycin as an anti-aging drug for the aging skin [1]). By now, there have been dozens of papers describing the therapeutic use of rapamycin (Sirolimus) in patients with such skin diseases as lymphatic malformations, vascular anomalies, Facial Angiofibroma and psoriasis [6–13]. These diseases were treated in children and young adults. In one study, topical rapamycin at low doses (0.003-0.015%) decreased facial angiofibromas in young adults. There was no systemic absorption of rapamycin (blood levels were <1.0 ng/mL) [13].

Returning to cellular senescence, signaling in the mTOR (Target of Rapamycin) pathway drives growth of cellular mass and sustains cell cycle progression. Cells grow and divide, balancing growth. But when the cell cycle is suddenly blocked by p16 or p21, mTOR drives growth-like conversion from reversible arrest (quiescence) to senescence [2,14]. In short, mTOR drives geroconversion [15]. Rapamycin and its analogs, as well as pan-mTOR inhibitors, suppress geroconversion, thereby maintaining cells in a young healthy state. Moreover, these drugs prevent loss of cells’ proliferative potential, which is considered a strict definition of senescence [2,15]. Geroconversion in stem cells leads to stem cells depletion [16,17]. mTOR-driven hypertrophy can be followed by atrophy at the end stages. Cellular hyperfunction eventually leads to cellular exhaustion and secondary functional decline [1].

Suppression of cellular senescence by rapamycin was demonstrated in numerous studies both in vivo and in vitro [18–30] and see for references [15]. In vitro, rapamycin slows conversion to senescence by approximately 3-fold [14]; it does not suppress it completely. Notably in that regard, in the most rapamycin-responsive mouse model of mitochondrial disease, rapamycin extends the maximum life span by nearly 3-fold [31].

Just as *in vitro* geroconversion is a continuation of growth, organismal aging is an unintended and harmful continuation of developmental growth post-development [1,32]. These messy quasi-programs inevitably lead to age-related diseases, which include conditions ranging from obesity, cancer and Alzheimer’s disease to skin spots, wrinkles and seborrheic keratoses. mTOR drives geroconversion, increasing cellular functionality (e.g., the senescence-associated secretory phenotype). It is noteworthy that this increase in cellular activity can cause secondary exhaustion, tissue damage and decreased of organ function; for example, hypertrophy may be followed by atrophy at later stages. In other words, age-related diseases and conditions initially caused by mTOR-driven hyperfunction eventually lead to organ damage and functional decline [1,33]. Similar quasi-programs were described even in the worm [34–36]. In sum, aging is an unintentional and harmful continuation of developmental programs, driven in part by mTOR. To be clear, mTOR activity does not need to increase with age, just keeping it at a level as high as during development is sufficient to cause disease. Despite its simplicity, this model accurately predicts that rapamycin will extend life and delay diseases. Indeed, since initial publications [18,37–39], numerous studies have confirmed that rapamycin extends lifespan in mice (see for references [40–44]).

In that context, it is predictable that rapamycin would slow skin aging. However, unless rapamycin reverses skin aging, not merely slow it, the effect would be difficult to document. This is because a patient cannot serve as a self-control (placebo control) unless rapamycin reverses aging, which would be easy to detect. This difficulty can be overcome, however, by comparing an untreated hand with a hand treated with topically applied rapamycin in the same subject. This is the approach taken by Chung et al. in their study, which found that treatment with rapamycin-containing cream improved skin photoaging and skin tone, decreased fine wrinkles, increased dermal volume, and reduced sagging of the skin [3]. These differences between treated and untreated hands were detectable after 4 months of the treatment [3]. Regrettably, the study excluded patients with diabetes, although the therapeutic effect would probably be more significant in diabetic patients, given that mTOR is overactivated in that disease. In addition, it is unclear whether rapamycin reversed skin aging and improved the skin or merely slowed the progression of skin aging. In the latter scenario, the difference between the treated and untreated hands is due to the progression of aging in the untreated hands. In combination with placebo/treatment, comparisons of specific abnormalities before and after treatment is also needed. Despite these open questions the study is remarkable [3].

As a cosmetic, rapamycin-containing cream may be applied to selected areas, like the hands and face, especially skin affected by age-related spots and pathologies. It should not be applied to the entire skin surface of the body. To affect the entire skin surface, systemic use of rapamycin would likely be a better option, as many manifestations of skin aging are probably due to systemic organismal aging and disease; skin aging is not an exclusively local process. And most importantly, systemic rapamycin use increases lifespan and decreases disease. This by itself is so important that solely topical use of rapamycin may seem insufficient. On the other hand, topical application of any drug is safer than systemic administration. Still, the best strategy in some cases may be simultaneous systemic and topical use of rapamycin in selected areas of the skin, especially areas where there are signs of aging marks. However, given that most doctors are fearful of systemic treatment with rapamycin [45], I expect that it will be topical use of rapamycin that becomes widespread, if regulatory hurdles can be overcome. Whether rapamycin cream should be a prescription treatment or an over-the-counter cosmetic will likely be a matter of debate.

## Patent Application Publication

https://patents.google.com/patent/WO2008022256A2/en
